# *In vitro* screening of antifungal activity of marine sponge extracts against five phytopathogenic fungi

**DOI:** 10.1186/2193-1801-3-629

**Published:** 2014-10-24

**Authors:** Belkassem El Amraoui, Majida El Wahidi, Aziz Fassouane

**Affiliations:** Laboratoire Contrôle Qualité en Bio-Industrie et Molécules Bio-Actives, Faculté des Sciences, Université Chouaib Doukkali, BP 20, El Jadida, 24000 Maroc; Université Ibn Zohr, Faculté Polidisciplinaire de Taroudant, Taroudant, 82000 Maroc

**Keywords:** Antifungal activity, Porifera, *Cynachirella*, Marine sponges, *Haliclona*

## Abstract

The aim of our research is the screening of extracts of marine sponges for their antifungal activity against phytopathogenic fungi. The *in vitro* screening of hydroalcoholic and organic extracts of ten marine sponges from Atlantic coast of Morocco against five phytopathogenic fungi (*Fusarium oxysporum* f.sp. *melonis*, *Fusarium oxysporum* f.sp*. radicis-lycopersici, F*usarium *oxysporum* f.sp. *ciceris*, *Botrytis cinerea* and *Penicillium digitatum*) showed that only two sponges (*Haliclona viscosa* and *Cynachirella tarentina)* are active against all phytopathogenic fungi studied.

## Introduction

Agriculture in Morocco is an important economic sector, with 40% of the population living on its revenues. The agricultural area is estimated to be 9.5 million hectares. Fungi are the main responsible agents for losses in agriculture and horticulture and can infect any part of the plant (Messiaen et al. 1991). The fight against these fungi is based on the use of chemical pesticides. However, chemical pesticides sprayed into the air or discharged into the soil can be harmful to the environment and to humans as well.

More than 15 000 natural products were isolated between 1965 and 2005 from marine organisms (Blunt et al. 2007). One of the main factors contributing to this trend is related to modern technology, and ocean biodiversity has become more accessible (Battershill et al. 2005).

Most marine invertebrates, which lack defence structures, have developed chemical defence systems in producing toxic secondary metabolites (Anderson et al. 1994; Aratake et al. 2009).

The sponges, which have a very primitive origin, adopted and developed a cemical very powerful defence (Sipkema et al. 2005) and are the source of many chemical compounds with various biological activities, including antitumor (Acosta and Rodriguez 1992; Carmely et al. 1989), antiviral (Carter and Rinehart Jr 1978) antialgal (Wright et al. 2011), anti-inflammatory (Randazzo et al. 2001), antiparasitic (Galeano et al. 2011; Kossuga et al. 2008), antibacterial (Ankisetty and Slattery 2012; El Amraoui et al. 2014) and antifungal activities (Clark et al. 2001; El-Amraoui et al. 2013; Sata et al. 1999). These compounds also show the chemical diversity, and are composed among others of unusual nucleosides (Bergmann and Feeney 1951; Wang et al. 2009), peptides (Sjogren et al. 2006), and fatty acids (Carballeira et al. 2007; Carballeira and Pagan 2001; Pham et al. 1999).

Sponges consist not only of sponge tissue but also of microorganisms, which represent 50% of their mass. So, is that the antifungal that is secreted by the sponge or a microorganism associated with this sponge? The isolation of microorganisms associated to sponges and screening of antifungal metabolites produced by these microorganisms may provide an answer.

In Morocco, the researches of the metabolism products of medicinal plants and other groups of terrestrial or marine organisms are intensified to explore the possible use of metabolites in different areas. Despite the richness and biodiversity of the Moroccan sea, invertebrates and algae from seabed are poorly studied.

In this study, we report the antifungal activity of ethanol and dichloromethane extracts of ten marine sponges collected from Coastal Atlantic of El Jadida (Morocco) to select the most active species, which could be utilized to purify antifungal compounds.

## Results and discussion

The identification of sponge species and their sampling sites are summarized in Table 1.

**Table 1:** **Sponge’s identification and their sampling sites**

Sampling sites	References	Sponges species
Site n°1	EM6	*Cliona celata*
Site n°2	EM8	*Cinachyrella tarentina*
Site n°3	EM7	*Ircinia oros*
	EM11	*Ircinia spinulosa*
	EM12	*Ircinia dendroides*
	EM13	*Haliclona mediterranea*
	EM14	*Haliclona viscosa*
Site n°4	EM5	*Axinella polypoides*
Site n°5	EM3	*Haliclona enamela*
	EM10	*Cliona viridis*

The geographical location and depth of sampling sites are shown in Figure 2.

The results of the screening of antifungal activity of sponge extracts against phytopathogenic fungi are summarized in Table 2.

**Table 2:** **Antifungal activity of the marine sponge extracts**

Marine sponges	Extract	Inhibition diameter (mm)				
		***FOM***	***FORL***	***FOC***	***BC***	***PD***
*Axinella polypoides*	B	-	-	-	-	-
	C	-	-	-	-	-
*Haliclona enamela*	B	-	-	-	-	-
	C	-	-	-	-	-
*Haliclona mediterranea*	B	-	-	-	-	-
	C	-	-	-	-	-
*Haliclona viscosa*	B	20	16	19	21	20
	C	17	14	17	20	18
*Cinachyrella tarentina*	B	-	-	-	-	-
	C	14	15	15	19	17
*Cliona celata*	B	-	-	-	-	-
	C	-	-	-	-	-
*Cliona viridis*	B	-	-	-	-	-
	C	-	-	-	-	-
*Ircinia dendroides*	B	-	-	-	-	-
	C	-	-	-	-	-
*Ircinia spinulosa*	B	-	-	-	-	-
	C	-	-	-	-	-
*Ircinia oros*	B	-	-	-	-	-
	C	-	-	-	-	-
DESOGERME SP VEGETAUX® (100 μg)		28	26	28	29	34

–: negative activity; *FOM: Fusarium oxysporum* f.sp. *melonis; FORL: Fusarium oxysporum* f.sp. *radicis-lycopersici; FOC: Fusarium oxysporum* f.sp. *ciceris*; BC: *Botrytis cinerea; PD: Penicillium digitatum.*

Among 20 extracts tested, only three extracts (15%), showed antifungal activity against the studied phytopathogenic fungi.

**Figure 1:**
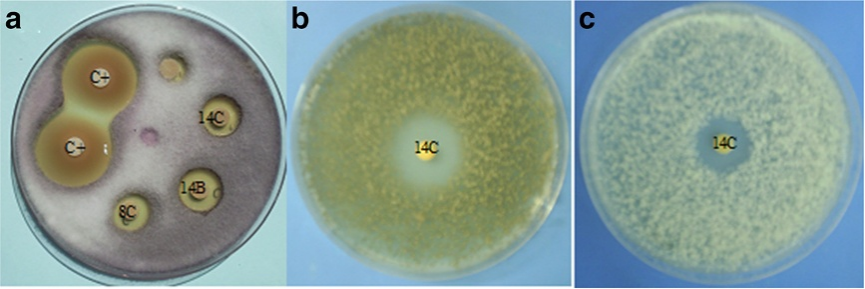
**Example of antifungal activity of extracts of** ***H. viscosa*** **and** ***C. tarentina*** **against** ***Fusarium oxysporum*** **f.sp.** ***melonis*** **(a),** ***Botritis cinerea*** **(b) and** ***Penicillium digitatum*** **(c).** (C+ : positive control (Desogerme sp); 8C: dichloromethane extract of *C. tarentina*; 14C: dichloromethane extract of *H. viscosa*; 14B: ethanol extract of *H. viscosa*).

Organic and hydroalcoholic extracts of *H. viscosa* exhibit antifungal activity whereas in *C. tarentina,* only the organic extract is active. This inhibition’s effect can be shown in Figure 1.

The sponge genus *Haliclona* is known for its high chemical various secondary metabolites with interesting biological activities (Faulkner 2002) including the antifungal (Barrett et al. 1996; Clark et al. 2001; El-Wahidi et al. 2013), antileishmanial (Dube et al. 2007), antioxidant (Regoli et al. 2004), cytotoxic (Fusetani et al. 1989) and other activities (Lakshmi et al. 2009; Randazzo et al. 2001).

Up to now, the researches conducted on *H. viscosa* led to the isolation of a number of alkaloids. Fusetani *et al*. 1989 have isolated two cytotoxic compounds, haliclamine A and B from *H. viscosa*. Volk and Kock 2003 have isolated viscosamine, then viscosaline in Volk and Kock 2004. Recently, two forms of viscosaline have been isolated (Schmidt et al. 2012). In addition to this, two other alkaloids, haliclamine C and D, were isolated from *H. viscosa* (Volk et al. 2004).

Lately, we have isolated a new product called haliscosamine from *H. viscosa* (El-Amraoui et al. 2013). This product is active against yeasts involved in human pathology. The chemistry of *Cinachyrella tarentina* sponge is rarely studied (El-Amraoui et al. 2010; El-Wahidi et al. 2011). This sponge was discovered in Italy (Pulitzer-Finali 1983), and now, we have collected this sponge from the Deauville beach, El Jadida, Morocco (El-Amraoui et al. 2010).

## Conclusion

Preliminary results have shown that Moroccan sponges constitute a potential source of compounds, which can be used for crop protection. *Haliclona viscosa* and *Cinchyrella tarentina* have an interesting antifungal potential. Thus, these sponges provide a potential source of antifungal compound to fight against plant diseases and should be investigated for isolation of this natural compound.

## Materials and methods

### Phytopathogenic strains

Strain of *Fusarium oxysporum* f.sp. *melonis* (FOM 20474 CECT) was obtained from Coleccion Espanola de Cultivos Tipo (Suárez-Estrella et al. 2007), *Fusarium oxysporum* f.sp*. radicis-lycopersici (FORL), F*usarium *oxysporum* f.sp. *ciceris* (*FOC*) and were obtained from the laboratory of Plant Pathology, Faculty of Sciences (El Jadida, Morocco), *Botrytis cinerea* (BC630) was obtained from biology-biochemistry department, Reims Champagne-Ardenne University, France and *Penicillium digitatum* (*PD001*), isolated from an infected orange, were used throughout this study.

### Sponge materials

**Figure 2:**
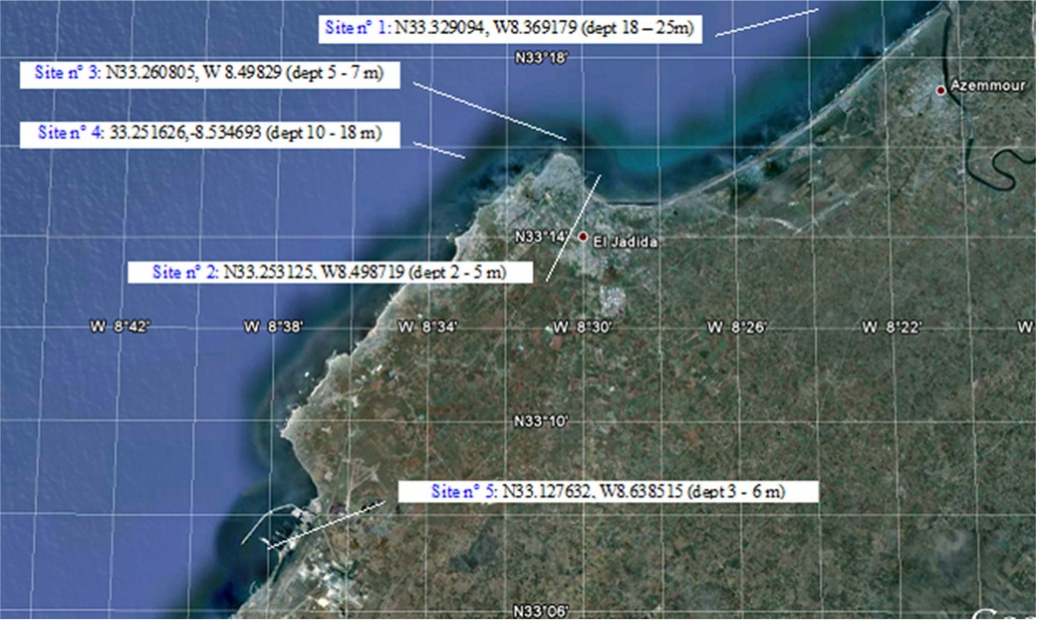
**Map showing the locations and depths of harvesting sponges.**

Ten marine sponges were collected from five sites of the littoral Atlantic coast of El Jadida (Morocco). Figure 2 shows the locations and depths of sampling sponges. All the sponges were identified by Dr. Maria-Jesús Uriz, Research Professor at the Centro de Estudios Avanzados de Blanes (CEAB) and Consejo Superior de Investigaciones Cientificas (CSIC) Spain by morphological characteristics and molecular methods (El-Amraoui et al. 2010). The collected materials were immediately frozen at −4°C for one night prior to extraction.

### Preparation of the extracts

Each sponge (100 g wet weight) was thawed, and extracted with ethanol (3 × 100 ml). The ethanol was evaporated at reduced pressure. The suspension was completed with sterile water to 100 ml and extracted with CH_2_Cl_2_ (3 × 100 ml).

The CH_2_Cl_2_ extract was dried on anhydrous sodium sulphate (Na_2_SO_4_), then filtered and concentrated at reduced pressure to give a **C** extract.

The aqueous phase was lyophilised and dissolved twice in absolute ethanol, then filtered and concentrated at reduced pressure to give a **B** extract (El-Amraoui et al. 2010).

### DESOGERME SP VEGETAUX®

DESOGERME SP VEGETAUX® (LAKORALE, Morocco), used in this study as a positive control, is an algaecide, fungicide and bactericide product used in Morocco both to remove algae, fungi and bacteria in irrigation systems and also to disinfect soil. It consists of 20 g/L of polyhexamethyle bioguanidine hydrochlorique, and 50 g/L of N-alkyl dimethyl benzyl ammonium chloride.

### *In vitro*antifungal activity

The antifungal activity was assessed *in vitro* by agar disc-diffusion test.

### Agar disc-diffusion test

This test uses Potato Dextrose Agar (PDA) as medium [Difco]. Conidial suspension was prepared from a 5-d-old fungal culture and adjusted with Malassez’s cellule in sterile water in order to obtain a final concentration of 10^5^conidia/mL. Each disk received 100 μg of sponge extract (20 μL of each extract at 5 mg/mL were added to each cellulose disc) and was applied on the test media which were previously inoculated with each test strain (El-Amraoui et al. 2010). Plates were first kept at 4°C for at least two hours to allow the diffusion of chemicals, and then incubated at 28°C. Inhibition zones were measured after 24 h of incubation (Galeano and Martınez 2007). Standard disks of DESOGERME SP VEGETAUX® (100 μg) served as positive antifungal control. All the assays were carried out in triplicate.
